# Relationship Between Intraocular Pressure and the True Rate of Functional and Structural Progression in the United Kingdom Glaucoma Treatment Study

**DOI:** 10.1167/iovs.66.1.32

**Published:** 2025-01-14

**Authors:** Giovanni Montesano, Alessandro Rabiolo, Giovanni Ometto, David P. Crabb, David F. Garway-Heath

**Affiliations:** 1 NIHR Biomedical Research Centre, Moorfields Eye Hospital NHS Foundation Trust and UCL Institute of Ophthalmology, London, United Kingdom; 2 City, University of London, Optometry and Visual Sciences, London, United Kingdom; 3 Department of Health Sciences, University Eastern Piedmont “A. Avogadro”, Novara, Italy; 4 Eye Clinic, University Hospital Maggiore della Carità, Novara, Italy

**Keywords:** glaucoma, progression, intraocular pressure, structure-function, visual field

## Abstract

**Purpose:**

To investigate the effect of average intraocular pressure (IOP) on the true rate of glaucoma progression (RoP) in the United Kingdom Glaucoma Treatment Study (UKGTS).

**Methods:**

UKGTS participants were randomized to placebo or Latanoprost drops and monitored for up to two years with visual field tests (VF, 24-2 SITA standard), IOP measurements, and optic nerve imaging. We included eyes with at least three structural or functional assessments (VF with <15% false-positive errors). Structural tests measured rim area (RA) with Heidelberg retina tomography (HRT) and average peripapillary retinal nerve fiber layer (pRNFL) thickness with optical coherence tomography (OCT). One eye of 436 patients (222 on Latanoprost) was analyzed. A Bayesian hierarchical model estimated the true RoP of VF and structural metrics, and their correlations, using sign-reversed multivariable exponential distribution. RA and pRNFL measurements were converted to a dB scale, matching the VF metric (mean deviation [MD]). The effect of average IOP on the true RoPs was estimated.

**Results:**

True RoP at the mean average IOP (17 mm Hg) was faster (*P* < 0.001) for VF-MD (−0.59 [−0.73, −0.48] dB/year) than HRT-RA (−0.05 [−0.07, −0.03] dB/year) and OCT-pRNFL (−0.08 [−0.11, −0.06] dB/year). The proportional acceleration of RoP per mm Hg increase was, however, not significantly different (smallest *P* = 0.15). Accounting for the structural floor-effect largely eliminated the differences in RoPs (smallest *P* = 0.25).

**Conclusions:**

VF appeared to deteriorate at a faster rate than structural measurements. However, this could be explained by the floor-effect from nonfunctional tissue. IOP induced a similar acceleration in RoP per mm Hg increase.

Glaucoma is a progressive optic neuropathy characterized by loss of visual field (VF) and associated structural changes of the optic nerve head (ONH). Elevated intraocular pressure (IOP) remains, at present, the only modifiable risk factor for the development and progression of glaucoma.^1^^–^^4^ IOP is therefore the main target of therapeutic intervention.

There is strong evidence that reducing IOP is effective in slowing glaucoma progression.^1^^,^^2^ In the United Kingdom Glaucoma Treatment Study (UKGTS),^1^ patients treated with latanoprost had a significantly lower risk of showing VF deterioration compared to the placebo arm. Despite this, many of the untreated patients (74%) did not reach the progression endpoint, whereas 15% of the treated patients progressed despite treatment.

Understanding the relationship between IOP and disease progression is made difficult by several factors. Part of the uncertainty is likely due to variable interindividual susceptibility to IOP. However, a large contribution comes from measurement imprecision. IOP is measured sparsely during clinical appointments, making it difficult to estimate the true pressure profile, even when dense phasing protocols are used.^5^^–^^8^ On the other hand, biomarkers of glaucoma progression, such as standard automated perimetry and imaging-derived metrics for ONH structure, are affected by noise, which confounds the estimation of the true rate of progression (RoP).^9^

We have recently shown that it is possible, with some assumptions, to deconvolve the distribution of true VF RoPs from the observed distribution of rates contaminated by noise and learning,^9^ using a hierarchical Bayesian model. The model can be reformulated to quantify the effect of specific variables, such as the IOP, on the distribution of true RoPs. This offers a more direct insight into the mechanism of glaucoma progression. In this work, we extend the model to quantify the effect of IOP on true glaucoma progression using both structural and VF measurements over time in patients from the UKGTS. The structural and functional measurements are modeled as a joint multivariable outcome, allowing the model to estimate the correlation between their true RoPs.

## Methods

The UKGTS was a two-arm, double-blinded, randomized clinical trial in which patients were allocated to receiving IOP-lowering drops (latanoprost) or placebo drops.^1^ Patients were followed-up for up to two years at regular intervals with VF tests, ONH imaging, and IOP measurements. Visits were planned at zero, two, four, seven, 10, 13, 16, 18, 20, 22, and 24 months. The study recruited 516 patients (258 in the treated arm). The main outcome of the trial was the risk of VF progression from baseline using a set of predetermined criteria, based on the Guided Progression Analysis.^1^ Details of the IOP, imaging, and VF tests are reported below. For both structural and VF tests, we only retained series that had at least three visits over at least six months. Note that eyes did not need to have sufficient samples for all metrics to be included. When both eyes were available, we selected the eye with worse baseline MD, following the prespecified statistical analysis plan.^1^

### VF Tests

VF tests were planned at each visit using a Humphrey Field Analyzer (Carl Zeiss Meditec, Dublin, CA, USA), 24-2 pattern, and a SITA-Standard thresholding strategy.^1^ The test was repeated twice at zero, two, 16, 18, and 24 months, for a total of 16 planned tests over the duration of the trial. For this analysis, we only included tests with a false-positive error rate ≤15%.^10^ We used the mean deviation (MD) for this analysis.

### Optic Nerve Head Imaging

Imaging with the Heidelberg retina tomograph (HRT; Heidelberg Engineering, Heidelberg, Germany) was planned for all patients. This imaging technique uses infrared light to perform a tomographic reconstruction of the retina and ONH surface. The reconstructed surface is then analyzed to derive metrics related to loss of neuronal tissue in the nerve.^11^^,^^12^ The most commonly used metric is the rim area (RA), which quantifies the area occupied by the neuro-retinal rim within the disc in an axial projection of the scan (in mm^2^).

Time domain optical coherence tomography (OCT) imaging was also performed for a subset of patients in centers where the device was available (Stratus; Carl Zeiss Meditec). This imaging technique uses infrared interferometry to obtain tomographic reconstructions of the reflectance of different retinal structures. Different layers can then be segmented and measured. The Stratus quantifies the circumpapillary thickness profile of the retinal nerve fiber layer (RNFL) at a fixed distance from the ONH center (3.46 mm radius). For the main analysis, we took the average of this peripapillary RNFL (pRFNL) thickness provided by the device.

Because many consecutive HRT and OCT scans were taken by the technician at each visit, these repeats were averaged to improve the quality of the measurement. Note that these consecutive same-visit measurements exhibited very high correlation and could not be used as independent observations because this greatly affected the estimation of the noise component in the hierarchical model (see later).

### IOP Measurements

IOP was measured at each visit using Goldmann applanation tonometry (GAT). At the first and final visit, a diurnal IOP curve was measured with GAT (every two hours, from 9 AM to 5 PM). For this analysis, we computed the average IOP for each eye. The diurnal values were collapsed into a single mean value before averaging across different visits.

### Progression Modeling

#### Base Model

The Bayesian hierarchical model used to estimate the distribution of true RoPs has been described in detail previously.^9^ The framework is the same as standard Gaussian hierarchical models (or linear mixed effect models, LMMs). Briefly, the model has two levels in the hierarchy, the population level and the patient level (assuming one eye per patient). The model estimates the intercept and slope of the change of a chosen index, such as the MD, over time. The patient level intercepts and slopes are modeled as samples from a random effect distribution, the parameters of which are estimated at the population level.

In our model, we assume that the indexes used to monitor glaucoma can only remain stable or become worse over time. A sign-reversed exponential distribution adequately describes the linear slopes obtained under this assumption, with a single parameter. We have described how the observed distribution would be contaminated by the distribution of measurement imprecision (noise).^9^ In the case of linear regression, this corresponds to a Gaussian distribution, with the standard deviation (SD) corresponding to the standard error of the slope. The resulting distribution is an exponentially-modified Gaussian (exGaussian) (i.e., the convolution of the Gaussian noise component and the underlying exponential distribution). In a Bayesian framework, the exGaussian distribution can be simply obtained by drawing samples from the exponential and Gaussian distributions and summing them to obtain the observed patient-level slopes. We have also shown that for VF MD, the mean of the noise distribution can capture the effect of learning (i.e., improvement in performance over time).

It is important to note that the true RoP for an individual eye cannot be estimated. This is because each observed RoP is calculated as the sum of a random draw from the exponential distribution of true rates and a random draw from the Gaussian noise distribution. Although the population (average) parameters for these distributions can be estimated, there are infinite pairs of random values that can result in the same observed RoP for an individual eye.

#### Extension to Multivariate Outcome

For our analysis, we extended the model to simultaneously estimate progression of HRT-RA, OCT-pRNFL and VF-MD. To compare these metrics on the same scale, rim area and pRNFL were converted into a decibel scale (i.e., 10*log_10_[rim area] and 10 × log_10_[pRNFL]). For individual metrics, the same hierarchical model can be used to estimate the exponential distribution of true RoPs of structural metrics. Because no learning is expected for structural metrics, the mean of the noise distribution was constrained to be zero for the HRT-RA and OCT-pRNFL RoPs. This assumption was verified by inspecting the plots of the residuals of simple linear regressions over time for the three metrics (see Supplementary Figure S1).

Medeiros et al.^13^ showed how the RoPs of the structural and VF metrics can be modeled jointly with a multivariate normal distribution for the random effects. The same reasoning can be applied to modeling the distribution of true RoPs. However, there is no straightforward way to model the correlation between multiple exponential distributions via a multivariate equivalent. One common approach is to use normal copula functions.^14^ With normal copulas, the correlation is modeled through a standard multivariate normal distribution. This distribution produces correlated samples, the marginal distributions of which are all standard normals (*mean* = 0, *SD* = 1). The marginals can then be converted into cumulative probability scores and then remapped to any arbitrary distribution, such as the exponential distributions of interest. This preserves the *rank correlation* between the copula marginal distributions and the target distributions. An example with simulated data is shown in Supplementary Figure S2. Note that the parameters for the exponential distributions can be estimated as in the univariate case. The correlation coefficients are the only free parameter for the normal copula, because its variances are set to one and its means are set to zero. The same process was followed for modeling the intercept. In this case, however, we used a simple multivariate normal distribution. We have shown that the distribution for the intercept could be approximated by an exGaussian.^9^ However, this choice did not greatly affect the estimates of the slopes while adding complexity to the model.

**Figure 1.**
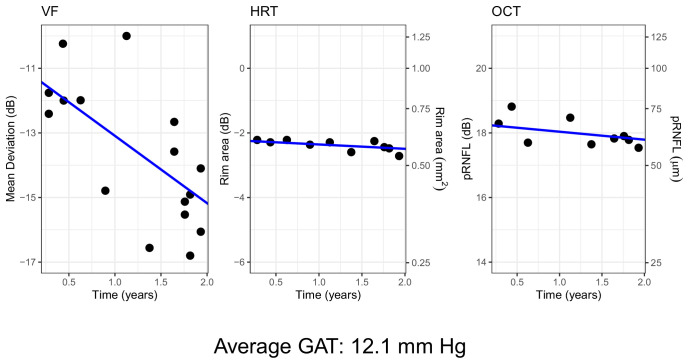
Example of fitting results for one eye. The *blue lines* represent the estimated trend for the observed rate of progression (random effect predictions). The data points are all reported in the dB scale used for the analysis. For the rim area and the mRFNL, the linear scale is reported as a secondary axis on the right. The vertical axes have been scaled to cover the same range of 8 dB, allowing a direct comparison of the slopes.

One important aspect to note is that the joint modeling of the slopes does not require that the different measurements be taken at the same visit. Moreover, it does not require that all patients have sufficient data for all metrics, because Bayesian computations can draw samples from the posterior distributions for missing data. Therefore all eyes with a sufficient number of valid tests/visits for at least one of the test (VF, HRT or OCT) were included in the final sample. An example of the fitting results for one eye (random effect predictions) from one of the models is shown in Figure 1.

#### Effect of Intraocular Pressure

**Figure 2.**
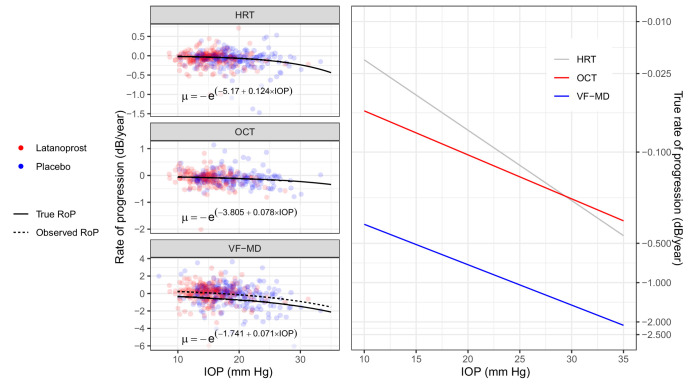
Effect of Goldmann IOP on the RoP of the VF-MD, HRT rim area, and OCT average pRNFL. Note that the HRT and OCT metrics are also converted to a dB scale for these calculations. The equations for the predicted mean of true RoPs (µ) are reported in the graphs. The observed RoP for VF-MD is different from the true RoP because of learning (see Table 2). The panel on the right shows a comparison of the IOP effect on the mean true RoP in log-scale, used for fitting the exponential component of the model. The *dots* represent simple linear regression rates in dB scale, color coded for the study arm.

The model allows the estimation of the effect of different factors on the predicted mean of the exponential distribution of true RoPs, such as treatment arm in the UKGTS or the effect of IOP across the range of IOP values (Fig. 2). In this analysis, we modeled these effects by having the mean of the exponential distribution continuously change following a log-normal regression model. This ensures positive values for the mean (a requirement for the model). For IOP, the model would simply be

$$log\left(\mu \right)=\beta_{0}+\beta_{1}*IOP$$

where *µ* is the sign-reversed mean of the exponential distribution of true RoPs. A similar model can be used to study the effect of the treatment arm assignment in the trial, instead of the IOP. The noise component of the slope was modeled independently for each type of measurement and was not related to IOP or arm assignment. The model for the intercept was identical but without a logarithmic link function for the mean. For fitting, IOP was centered on the average value and normalized by dividing by its standard deviation. In Figure 2, the IOP model prediction is superimposed to slopes estimates from simple linear regressions for the individual eyes. Note that the model cannot quantify the true RoP for each eye.^9^ In the tables, the effect of IOP is reported as a proportional (percentage) effect on the original scale of true RoP (in dB).

#### Supplementary Analyses

Additional analyses were performed to quantify the effect of specific scaling and mapping aspects that could have impacted the results.

##### Arithmetic Mean for VF

The MD is a weighted average of total deviation values^15^ (i.e., a weighted geometric mean of age and eccentricity corrected values). In contrast, the dB values for the structural metrics were calculated by taking the logarithm of the arithmetic mean of the linear values. Supplementary analyses were conducted by taking the logarithm of the mean of the anti-logged sensitivity values in the test, MLS = 10*log_10_ (mean linear sensitivity). Sensitivity was divided by 10 prior to anti-logging, to invert the dB calculation.

##### Structural Floor Effect

Structural measurements suffer from a floor effect, whereby nonneural and neural nonfunctional tissue offset the minimum value for the structural metrics. This means that even for very advanced disease, the measured thickness will be far from 0. This can introduce distortions when taking the logarithm. For example, a change from 100 µm to 70 µm would translate to a log_10_ difference of 0.15 (30% reduction). However, quantifying the actual change would require subtracting the floor, 45 µm in some reports,^16^ from both measurements before performing the calculation. This would translate to a difference in log_10_ scale of 0.34 (54%). Therefore a dB rate for structure is expected to be slower without considering the floor. We performed a supplementary analysis to estimate the magnitude of this effect. A precise calculation would, however, require estimating a personalized floor to be subtracted before taking the logarithm. We instead used an approximation by quantifying the average floor for each metric as the average OCT-pRNFL thickness and HRT-RA for measurements corresponding to VF tests with a mean total deviation ≤−15 dB, within a time window of ±3 months. Some eyes with advanced damage would however have structural measurements below this average floor. To avoid negative values, a different floor was calculated for each one of these eyes as the 90% of the smallest value in the series. The IOP model was fitted with these floor-corrected measurements, using both MD or MLS (see above).

##### Matched Structure-Function Mapping

For our analysis, we focused on global metrics of structural and functional damage. However, the structural metrics quantify the tissue around the whole circumference of the ONH. In contrast, the central retina is overrepresented in the 24-2 VF test. We performed supplementary analyses to estimate the impact of this discrepancy. We have fitted the IOP model using data from the central VF clusters (Garway-Heath clusters 2, 3 and 4; i.e., superior-paracentral, macular, and inferior paracentral), the average OCT-pRNFL thickness for clock-hours 7 to 11 (in right eye orientation; i.e., the temporal RNFL) and the sum of the rim-areas of the inferior-temporal, temporal and superior-temporal nerve sectors from the HRT. The VF data were modeled using either a simple average of the sensitivity values corresponding to the central clusters (a surrogate of MD) or the MLS (see above) for the same locations. Floor-corrected calculations were also performed, recalculating the floor effect of the nasal ONH tissue and using the mean total deviation derived from the central clusters.

#### Bayesian Computation

We used JAGS and the package runjags for R (R Foundation for Statistical Computing, Vienna) to run Markov Chain Monte Carlo sampling of the posterior distributions. We used two parallel chains with a thinning interval of 100 samples and a burn-in of 5000 samples. We monitored for all population-level parameters and we considered the chains to have converged when the Gelman-Rubin diagnostic metric was < 1.05 (minimum of 4000 samples per chain after thinning). The posterior samples from the two chains were merged and used to calculate 95% credible intervals (95% CIs) and a Bayesian metric similar to a frequentist two-sided *P*-value (*P*) as described by Makowski et al.^17^ and used in previous analyses by our group.^18^^,^^19^

## Results

### Patients’ Cohort

Summary statistics for the sample of eyes included in the analysis are reported in Table 1. As previously explained, individual eyes only needed eligible series for at least one of the functional or structural metrics. In total, 436 eyes were analyzed. Of these, 313 had sufficient measurements for all metrics, 98 for VF and HRT only, 17 for VF and OCT only, two for OCT and HRT only, and six for VF only.

**Table 1.** Sample Demographics

	Visual Field		HRT		OCT	
	Placebo, *N* = 213	Latanoprost, *N* = 221	Placebo, *N* = 199	Latanoprost, *N* = 214	Placebo, *N* = 160	Latanoprost, *N* = 172
Age (years)	67 (59, 74)	66 (60, 73)	66 (59, 73)	66 (60, 72)	66 (59, 74)	66 (60, 72)
Sex						
Female	107 (50%)	104 (47%)	101 (51%)	101 (47%)	78 (49%)	74 (43%)
Male	106 (50%)	117 (53%)	98 (49%)	113 (53%)	82 (51%)	98 (57%)
Race						
Black-African	4 (1.9%)	4 (1.8%)	3 (1.5%)	4 (1.9%)	3 (1.9%)	4 (2.3%)
Black-Afro-Caribbean	6 (2.8%)	3 (1.4%)	6 (3.0%)	3 (1.4%)	6 (3.8%)	3 (1.7%)
Indian Subcontinent	4 (1.9%)	9 (4.1%)	4 (2.0%)	8 (3.7%)	3 (1.9%)	8 (4.7%)
Other	6 (2.8%)	4 (1.8%)	6 (3.0%)	4 (1.9%)	6 (3.8%)	2 (1.2%)
White	193 (91%)	201 (91%)	180 (90%)	195 (91%)	142 (89%)	155 (90%)
BCVA (logMAR)	0.00 (−0.08, 0.18)	0.00 (0.00, 0.18)	0.00 (−0.08, 0.18)	0.00 (−0.08, 0.18)	0.00 (−0.08, 0.18)	0.00 (0.00, 0.18)
Average baseline GAT IOP (mm Hg)	19.5 (16.0, 22.0)	19.5 (16.0, 22.5)	19.5 (16.0, 22.0)	19.5 (16.0, 22.5)	19.5 (15.3, 22.8)	19.3 (16.0, 22.5)
Average follow-up GAT IOP (mm Hg)	18.1 (15.9, 21.4)	15.1 (13.5, 17.0)	18.1 (15.9, 21.3)	15.1 (13.5, 17.1)	18.6 (14.9, 22.1)	14.9 (13.2, 16.9)
Number of VF tests	14 (10, 16)	16 (11, 17)	9 (7, 11)	10 (7, 11)	9 (7, 11)	10 (7, 11)
Follow-up (years)	1.85 (1.34, 2.20)	2.10 (1.65, 2.22)	1.86 (1.42, 2.20)	2.06 (1.68, 2.21)	1.86 (1.34, 2.20)	2.05 (1.64, 2.20)
Baseline mean deviation (dB)	−3.3 (−5.6, −1.9)	−3.3 (−5.1, −1.9)				
Baseline pattern standard deviation (dB)	3.8 (2.5, 6.6)	3.8 (2.4, 6.1)				
HRT rim area (mm^2^)			0.94 (0.71, 1.19)	0.87 (0.68, 1.14)		
Average peripapillary RNFL thickness (µm)					74 (62, 83)	74 (66, 83)

MAR, minimum angle of resolution.

### Effect of Treatment on the True Rate of Progression

When the effect of the study arm assignment was modeled in isolation, there was a significant difference in the true RoP for the VF-MD, but not for the structural metrics (Table 2). The estimated average true RoP was faster for VF-MD than for the structural metrics (*P* < 0.001). The RoP for HRT-RA was significantly slower than OCT-pRNFL in the latanoprost arm (*P* = 0.007). There was a positive correlation for the true RoP between VF-MD, HRT-RA and OCT-pRNFL (Table 2). As expected, there was a significant learning effect in the RoP of the VF-MD. This was assumed to be the same for both arms, to maximize statistical power. The term baseline indicates the estimated mean value of the three metrics in dB (VF-MD, HRT-RA, OCT-pRNFL) at the intercept (i.e., when time = 0). In linear scale, the estimated RoP for OCT-pRNFL was −1.42 [−1.95, −0.88] µm/year and −1.79 [−2.37, −1.22] µm/year in the latanoprost and placebo arm, respectively (linear mixed effect model, *P* = 0.345). The estimated RoP for HRT-RA was instead −0.011 [−0.015, −0.007] mm^2^/year and −0.013 [−0.018, −0.009] mm^2^/year in the latanoprost and placebo arm, respectively (*P* = 0.446).

**Table 2.** Model Estimates Comparing for the Two Trial Arms

	Placebo	Latanoprost	*P*
VF-MD			
Baseline (dB)	−3.924 [−4.332, −3.499]	−3.795 [−4.211, −3.402]	0.659
True RoP (dB/year)	−0.778 [−0.971, −0.628]	−0.507 [−0.652, −0.384]	0.004
Learning (dB/year)	0.591 [0.472, 0.707]		<0.001^*^
HRT–RA			
Baseline (dB)	−0.433 [−0.678, −0.173]	−0.775 [−1.012, −0.531]	0.051
True RoP (dB/year)	−0.068 [−0.104, −0.042]	−0.025 [−0.079, −0.007]	0.105
OCT-pRNFL			
Baseline (dB)	18.638 [18.517, 18.779]	18.702 [18.575, 18.827]	0.501
True RoP (dB/year)	−0.089 [−0.134, −0.059]	−0.086 [−0.119, −0.059]	0.881
**Correlation coefficient (True RoP)**		
VF-HRT, estimate [95% credible interval]	0.608 [0.186, 0.927]
VF-OCT, estimate [95% credible interval]	0.656 [0.387, 0.967]
HRT-OCT, estimate [95% credible interval]	0.614 [0.087, 0.985]

The baseline represents the estimated mean value when time = 0. The true RoP is the predicted mean of the exponential component of the distribution of slopes for each arm.

^*^For difference from 0 dB/year.

### Effect of IOP on the True Rate of Progression

The effect of the average GAT IOP on the true RoP is shown in Figure 2 and Table 3. For ease of interpretation, the model coefficients are reported as the estimated true RoP at the average IOP (17 mm Hg) and the percentage change per mm Hg (the slope in log-scale). The model coefficients are reported in Figure 2. The effect of IOP was significant for VF-MD (*P* < 0.001), HRT-RA (*P* = 0.001) and OCT-pRNFL (*P* = 0.006). Similarly to the model estimating the effect of the treatment arm, the estimated average true RoP was faster for VF-MD than for the structural metrics (*P* < 0.001) and there was a positive correlation for the true RoP between VF-MD, HRT-RA and OCT-pRNFL (Table 3). Interestingly, the percentage change in true RoP per mm Hg was similar for all three metrics (smallest *P* = 0.15 for pairwise comparisons).

**Table 3.** Model Estimates for the Effect of Intraocular Pressure on the True RoP

	Baseline at 17 mm Hg (dB)	Change in Baseline (dB) per mm Hg	True RoP at 17 mm Hg (dB/Year)	% Change in RoP per mm Hg	Learning (dB/Year)
VF-MD	−3.86 [−4.14, −3.56]	−0.02 [−0.08, 0.05]	−0.59 [−0.73, −0.48]	7.38 [4.10, 10.97]	0.57 [0.45, 0.69]
HRT-RA	−0.61 [−0.78, −0.44]	−0.03 [−0.07, 0.02]	−0.05 [−0.07, −0.03]	13.21 [5.43, 21.73]	—
OCT-pRNFL	18.67 [18.58, 18.76]	−0.03 [−0.05, −0.01]	−0.08 [−0.11, −0.06]	8.07 [2.29, 13.85]	—
**Correlation Coefficient (True RoP)**	
VF-HRT	0.35 [−0.17, 0.85]
VF-OCT	0.63 [0.26, 0.95]
HRT-OCT	0.42 [−0.14, 0.96]

The baseline represents the estimated mean value when time = 0; this was also allowed to vary by average IOP.

A second version of the model was also estimated, setting the learning effect for VF-MD to 0 dB/year, as for the structural metrics. This was done to compare the average difference in RoP between VF-MD and structural metrics under different model designs. With this version of the model, the estimated true RoP at the average IOP was similar and not significantly different between VF-MD and structural tests, but the proportional effect of IOP was much faster for VF-MD (Supplementary Table S1).

An additional version of the model was tested, to take into account potential differences in the IOP effect between the two arms of the trial (with the use of interactions). There was no significant difference in the effect of IOP between eyes in the placebo or latanoprost arm (Supplementary Table S2).

### Supplementary Analyses

The numerical results of these analyses are reported in detail as supplementary material. Fitting the model using MLS instead of MD as a VF metric led to a slower estimate of VF RoP at the average IOP (−0.28 [−0.38, −0.20] dB/year, Supplementary Table S3), which was, however, still significantly faster than the structural metrics (*P* < 0.001). Similar results were obtained by restricting the analysis to the temporal ONH (nasal VF), as shown in Supplementary Tables S4 and S5, with the MS still showing a significantly faster RoP compared to structure (largest *P* = 0.007 for pairwise comparisons).

**Figure 3.**
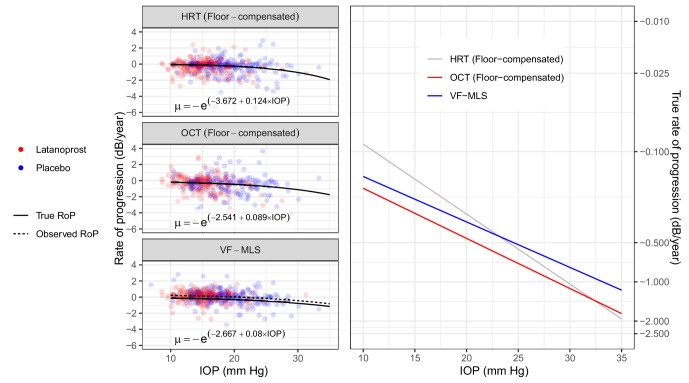
Effect of Goldmann IOP on the RoP of the visual field mean linear sensitivity (in dB scale) and on the floor-compensated HRT rim area and OCT average pRNFL, also converted to a dB scale. The equations for the predicted mean of true RoPs (µ) are reported in the graphs. The observed RoP for VF-MD is different from the true RoP because of learning. The panel on the right shows a comparison of the IOP effect on the mean true RoP in log-scale, used for fitting the exponential component of the model. The *dots* represent simple linear regression rates in dB scale, color coded for the study arm.

A substantially faster RoP for the structural metrics was instead obtained after compensating for the floor effect. The average floor was 0.70 mm^2^ for HRT-RA and 51.4 µm for OCT-pRNFL. The VF RoP was still significantly faster than the structural metrics (largest p = 0.002) when using MD (Supplementary Table S6). However, all parameters become very similar, and not significantly different (smallest p = 0.25 for pairwise comparisons) when performing the calculations with MLS (Supplementary Table S7). This result is also shown in Figure 3. Similar results were obtained when analyzing the temporal ONH and MLS for the nasal VF (Supplementary Table S8). In this case, the average floor was 0.22 mm^2^ for HRT-RA and 46 µm for OCT-pRNFL.

## Discussion

In this work, we used a Bayesian hierarchical model to estimate the effect of the average IOP on the true rate of functional and structural progression in glaucoma. Estimating the effect on the true RoP allows a more direct interpretation of the results, because it reduces the confounding effect of measurement noise and learning for functional metrics. The distribution of true RoPs was assumed to follow a sign-reversed (negative) exponential distribution, following previous work.^9^ The noise and learning effect were modeled by a Gaussian distribution. We modeled the correlations among the exponential distributions of true RoPs for VF and structural metrics, while maintaining independence for the noise distributions. The mean of the noise distribution modeled the learning effect for VF.^9^^,^^20^ This is particularly relevant for data from UKGTS because all the patients were recently diagnosed at the time of enrolment and therefore relatively naïve to VF testing.^1^ In our analysis, we transformed the structural metrics into a dB scale to improve the comparability of the different metrics and their change over time. This was preferred over a linearization of the MD to align with previous implementations of the Bayesian model^9^ and to prevent the amplification of noise that would derive from exponentiating a metric measured in a logarithmic scale. Decibel transformation of structural metrics has been used in previous work on structure-function correlations.^21^^,^^22^ It has the advantage of describing the thinning of neural tissue in terms of proportional change. This has been suggested as a better way to describe axonal loss from glaucoma or aging.^21^ Harwerth et al.^22^ reported an OCT-pRNFL thinning of 0.27%/year, corresponding to 0.3 µm/year. Percentage change can be derived from our results as (10^dB Rate/10^ − 1) * 100. For the OCT-pRNFL thickness at the average IOP, the true dB RoP was −0.08 dB/year. This corresponds to 1.86%/year, seven times faster than the proportional loss from normal aging. The average linear RoP in our cohort, calculated with a linear mixed model, was −1.59 [−1.98, −1.20] µm/year, more than five times the average linear loss from aging.

The VF-MD was the only metric able to identify a significant difference between the two arms of the trial (Table 1). We also found that the functional progression measured with MD was faster than structural progression, despite a generally moderate correlation among their true RoP (Tables 2, 3). There are various explanations for this result. Previous evidence by Gardiner et al.^23^ has shown that true functional progression precedes structural changes measured by spectral-domain OCT. Intuitively, this can be explained by the fact that retinal ganglion cells and their axons, as they are damaged by glaucoma, become dysfunctional before their death and subsequent efferocytosis.^23^ However, this intuitive picture would inevitably be confounded by a mixture of axonal death and dysfunction and might not fully explain our findings.

One potential source of disagreement comes from the fact that VF metrics mainly capture the functional response of the central-temporal retina, whereas 360° structural measurements around the ONH would be influenced by axons from the entire retina. We have addressed this issue in our supplementary analyses (Supplementary Tables S4, S5) by fitting the IOP with data from the temporal ONH and the nasal VF (i.e., by matching the structure-function topography). This, however, did not explain the differences observed between structural and functional RoPs.

The discrepancy between structural and functional RoPs might also be partially explained by the scaling differences derived from using the geometric mean (i.e., mean of log_10_-values) to calculate MD (a weighted average of age- and eccentricity-corrected total deviation values^15^) as opposed to the logarithm of the mean of linear uncorrected values, used for structure. We addressed this by fitting the model using the MLS for VF (Supplementary Table S3). The MLS RoP was slower but still significantly faster than the structural metrics.

The most likely explanation for our findings is that our analysis, despite matching the scale by transforming all metrics into a logarithmic dB measure, does not directly address the differences in effective dynamic range. As previously explained, structural metrics would be affected by a measurement floor introduced by the presence of dysfunctional and nonfunctional tissue, which would become more and more relevant as the disease progresses.^16^^,^^24^ In linear scale, the floor-effect is not expected to be large for initial glaucoma damage and for a follow-up limited to two years.^25^ However, this can affect the quantification of percentage loss in neural tissue at any level of damage (see Methods), because the structural floor offsets the assumed zero-value when taking the logarithm of the structural measurements. This is supported by our supplementary analyses, reported in Supplementary Tables S6, S7 and S8 and Figure 3, which show that floor-compensated structural metrics have RoPs that are similar to MLS. Taken together, these results indicate that loss of structure and function are likely to happen at a similar rate in response to IOP in glaucoma, when appropriate scaling and measurements floor are considered. One limitation of this analysis is that we could not calculate a personalized floor for individual eyes in the analysis, relying instead on a combination of average floor-effect estimates and the minimum values for eyes below the average floor (see Methods). Future research will focus on obtaining personalized estimates of the structural floor which would allow a more precise estimation of the effective dynamic range of structural metrics.

We also recommend caution in the interpretation of the results for the structural metrics, because the data collected during the UKGTS relied on now superseded imaging technology. This is particularly relevant for the model used for this analysis, which aims to distinguish the true RoP from the noise. This problem can be interpreted as an attempt to “deconvolve” the distribution of true RoPs from the distribution of noise. Deconvolution is generally an ill-posed problem because it can be impossible to solve when the true signal is overwhelmed by a large amount of noise. These findings will therefore need confirmation from studies employing more accurate measurements from modern imaging modalities, such as spectral-domain OCT. Another possibility would be to use super-resolution algorithms to improve the image quality of the data from UKGTS.^26^ Such an approach has shown to be effective in improving the statistical power of measurements obtained from Stratus OCT images^26^ and its application will be the objective of future work. It should, however, be noted that data from UKGTS provide the rare opportunity to investigate the effect of IOP on structural and functional progression in a cohort that is not contaminated by treatment escalation. This is unlikely to be the case in more modern trials or clinical dataset because of ethical concerns, making our results an important contribution to the understanding of the mechanisms of disease progression. Moreover, most of the seminal work on structure-function relationship in glaucoma was based on data from TD-OCT,^16^ meaning that our results can be directly compared with previous literature.

The percentage change in true RoP per mm Hg was very similar and not significantly different between the functional and structural metrics (Fig. 2; Table 3), indicating a similar proportional acceleration of the RoP per unit increase in pressure. It is interesting to note that we could not find any significant difference in the effect of GAT IOP on the true RoP between the two arms of the trial, indicating that most of the treatment effect was explained by the measured difference in IOP (Supplementary Table S2). However, it should be noted that the point estimates, especially for the effect of IOP on VF-MD progression, were substantially different in magnitude. This might point to a lack of power in identifying a differential effect rather than a true lack of difference. Analyses with larger datasets might be able to elucidate this aspect further.

We also evaluated the effect of specific implementations of the model. In fact, in our main analysis, we improved stability of the fitting by assuming no learning effect for the structural metrics. This has the advantage of maximizing the chance of measuring the true RoP from noisy data, such as those provided by the HRT or the Stratus OCT. However, this additional free parameter for the MD model can effectively act as a bias, positive in the case of learning. This could have, in turn, forced a more negative mean for the exponentially distributed true RoP. Implementing learning for the structural metrics led to unstable fitting results that did not achieve convergence. Setting the learning for VF-MD to zero, as for the structural metrics, resulted in a similar RoP for VF-MD and structural tests at the mean average IOP, but a much larger proportional effect of IOP on the VF-MD RoP (Supplementary Table S1). Although assuming no perimetric learning for naïve patients is likely incorrect, this change in the results should be borne in mind when generalizing the finding of this study. Additional insight might come from analyzing structural data obtained through better imaging and longer VF series, in which the effect of learning could be removed by eliminating earlier tests.^9^^,^^20^

In our analysis, similarly to previous investigations,^19^^,^^27^ we have summarized the effect of IOP by taking its average value over the entire follow-up period. However, IOP varies over time and its average might not fully capture the effect of IOP on progression. Other authors have proposed approaches to model IOP as a time-varying covariate.^28^ This would substantially increase the complexity of the model, and would require assumptions on the time lag between IOP variation and effect on progression. Some authors have speculated that fluctuations in IOP might have, on their own, an impact on progression.^28^^–^^31^ In contrast, a recent investigation by Rabiolo et al.^32^ using these same data from UKGTS showed that IOP fluctuations have no significant effect on the RoP, once the effect of average IOP is taken into account. Similar results were obtained in the Early Manifest Glaucoma Trial.^33^ Differently from clinical practice and other randomized clinical trials, IOP in the UKGTS was not managed to achieve a target pressure.^1^ This means that drastic changes in IOP are unlikely to occur as a result of reactive clinical management. Future work will focus on integrating the effect of dynamic changes in IOP for cohorts where larger changes in pressure are more likely to occur.^34^^,^^35^

**Figure 4.**
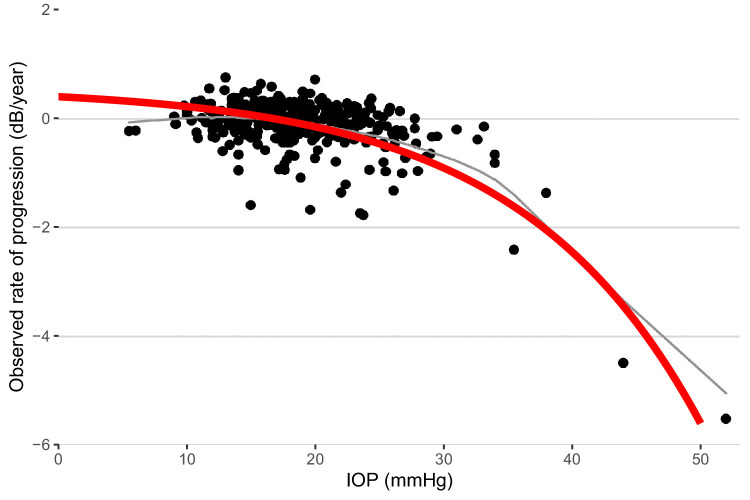
The prediction for the observed rate of visual field progression obtained from our model, fitted to the UKGTS cohort, overlaid on the data from Medeiros et al.^27^

By construction, our model predicts an exponential acceleration of the RoP with increasing average IOP, producing a curvilinear relationship (Fig. 2). Such a relationship has been described by Medeiros et al.^27^ Their cohort contained examples of more extreme cases with high IOPs and fast RoPs, which would be useful to test the predictions of our model. Figure 4 shows our prediction for the observed RoP (the same as the dashed line in Fig. 2) overlaid on the original plot from Medeiros et al.^27^ Our model not only appears to describe their observations but also offers a good prediction of the extreme cases beyond the range of IOPs in the UKGTS cohort (99th percentile for GAT: 28 mm Hg). Note that this result was achieved without requiring additional parameters in the model, such as the quadratic term used by Medeiros et al.^27^

Clearly, the spread of the observations around the predicted mean is large. Additional work will need to focus on refining the predictive ability of these models, possibly with the integration of other clinical, demographic, and genetic data or by modeling pointwise data. However, it should be kept in mind that, in the exponential distribution assumed for the true RoP, the expected standard deviation is equal to its mean. Other distributions such as a log-Normal or a Gamma, might be required to decouple the estimated variance from the predicted mean. These would, however, require the estimation of an additional parameter. The exponential distribution was chosen for the true RoPs because of its simplicity, stability during fitting and because we could not find evidence supporting more complex distributions when testing this model in a larger dataset of long VF test series^9^. However, this could, again, be a consequence of perimetric noise compromising the identification (deconvolution) of a more accurate distribution to describe the true RoPs. It is not excluded that the estimation of such a distribution will only be achieved when more precise tests will become available to monitor progression or by constraining the model with additional information about individual patients.

In conclusion, our results show that a joint model can be used to estimate the true RoPs of structural and functional metrics and their correlation in glaucoma patients, as well as their relationship with the average IOP. Functional progression appeared faster than structural progression. This disagreement likely derives from the effect of measurement floor in structural metrics, which appears to introduce fundamental differences in the relative magnitude of structural and functional changes. However, limitations of the modeling approach and especially of the precision of the imaging methods used to collect structural data should also be borne in mind when considering this disagreement. Additional studies should focus on elucidating this relationship further by evaluating patients with a wider range of IOP, longer follow-up time and more precise structural measurements with personalized estimates of their structural measurement floor.

## Supplementary Material

Supplement 1
