# Supplementary material for: Relationship Between Intraocular Pressure and the True Rate of Functional and Structural Progression in the United Kingdom Glaucoma Treatment Study
Source: Invest Ophthalmol Vis Sci. 2025 Jan 14;66(1):32. doi: 10.1167/iovs.66.1.32 (PMC11737456; doi:10.1167/iovs.66.1.32)
Supplement: Supplement 1 [file iovs-66-1-32_s001.pdf]

Supplementary material

***Supplementary figures* 1**

***Main model with no perimetric learning* 2**

***Main model estimates by treatment arm* 3**

***Main model estimates with perimetric Mean Linear Sensitivity* 3**

***Models for the temporal optic nerve/nasal visual field* 4**

With perimetric Mean Sensitivity (mean of dB sensitivity values) 4

With perimetric Mean Linear Sensitivity 4

***Models with floor-compensated structural metrics* 5**

With perimetric Mean Deviation 5

With perimetric Mean Linear Sensitivity 5

With matched structure-function mapping (temporal optic nerve, nasal visual field)..... 6

Supplementary figures

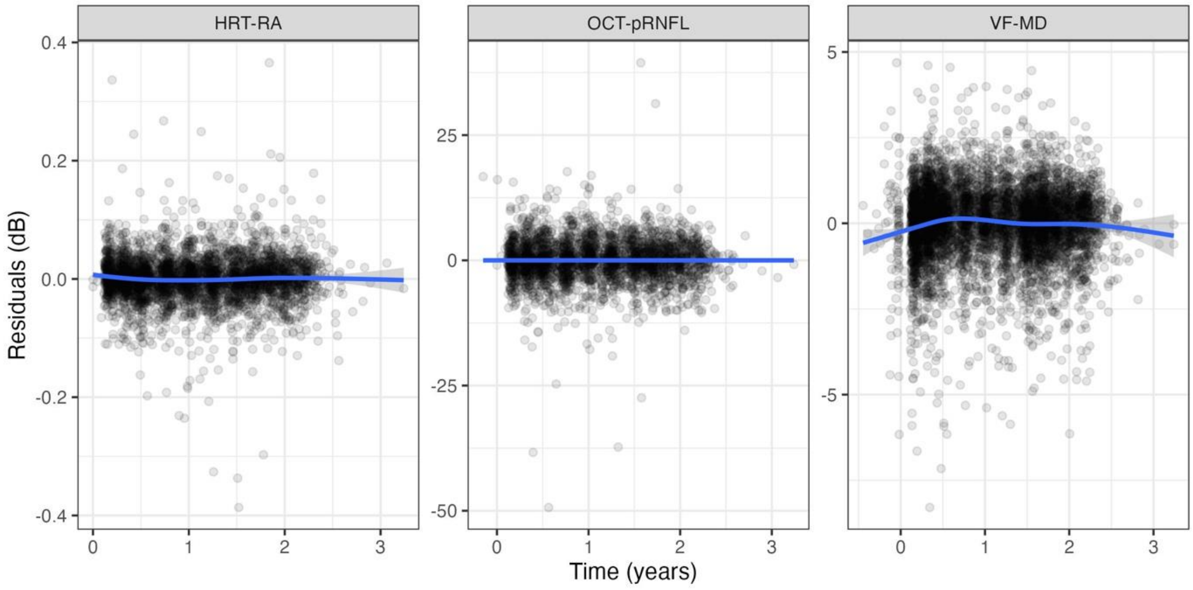

Supplementary Figure 1. Residual plot for the structural and VF metrics. The residuals for VF-MD show a positive trend over the first year of follow-up. This trend is not evident in the HRT-RA or the OCT-pRNFL. VF = Visual Field; MD = Mean Deviation; HRT = Heidelberg Retinal Tomograph; RA = Rim Area; OCT = Optical Coherence Tomography; pRNFL = peripapillary Retinal Nerve Fibre Layer.

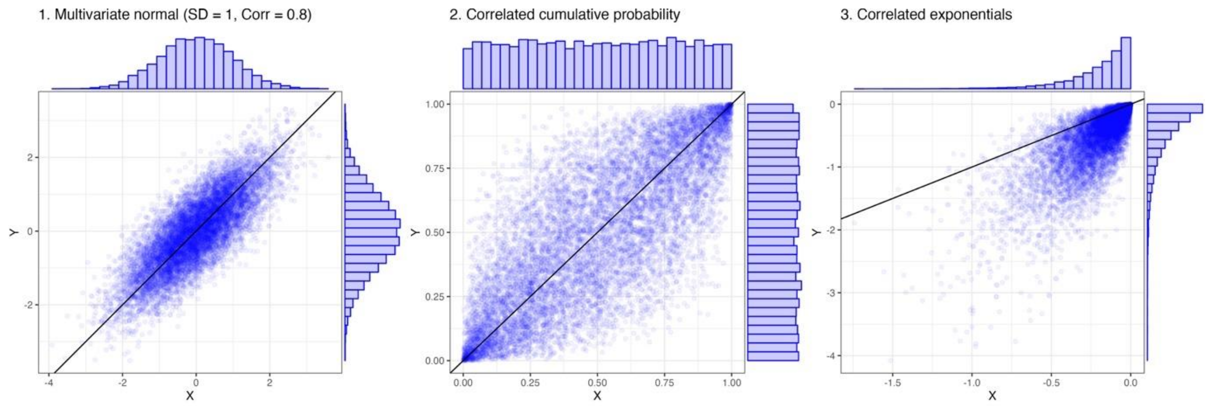

Supplementary Figure 2. Simulated example of how to draw from correlated exponential distributions using a normal copula. Correlated standard normal samples (**Panel 1**) are converted to normal cumulative probability values (**Panel 2**). These are then transformed into correlated exponential using inverse exponential cumulative distribution functions, preserving rank correlations among samples (**Panel 3**). The diagonal solid line is the identity line. In the example, the correlated marginal exponentials have different mean values.

Main model with no perimetric learning

GAT	Baseline at 17 mmHg (dB)	Change in baseline (dB) per mmHg	True RoP at 17 mmHg (dB/year)	% Change in RoP per mmHg
**VF-MD**	-3.81 [-4.10, -3.51]	-0.07 [-0.36, 0.22]	-0.05 [-0.12, -0.02]	36.24 [22.98, 51.20]
**HRT-RA**	-0.61 [-0.78, -0.44]	-0.11 [-0.29, 0.06]	-0.05 [-0.07, -0.03]	14.65 [6.39, 23.31]
**OCT-pRNFL**	18.67 [18.58, 18.76]	-0.13 [-0.22, -0.04]	-0.08 [-0.11, -0.06]	8.80 [2.36, 15.51]

**Correlation coefficient (True RoP)**		
**VF-HRT**	**VF-OCT**	**HRT-OCT**
0.46 [-0.15, 0.90]	0.48 [-0.26, 0.99]	0.42 [-0.16, 0.96]

Supplementary Table 1. Model estimates for the effect of intraocular pressure on the true rate of progression (RoP) when learning is set to 0 dB/year for VF. The % Change in RoP per mmHg was significantly larger for VF-MD compared to HRT-RA (p = 0.004) and OCT-pRNFL (p = 0.014). GAT = Goldman Applanation Tonometry; VF = Visual Field; MD = Mean Deviation; HRT = Heidelberg Retinal Tomograph; RA = Rim Area; OCT = Optical Coherence Tomography; pRNFL = peripapillary Retinal Nerve Fibre Layer; IQR = Interquartile Range.

Main model estimates by treatment arm

GAT	Placebo	Latanoprost	p
**VF-MD**			
Baseline at 17 mmHg (dB)	-3.86 [-4.31, -3.42]	-3.74 [-4.19, -3.29]	0.706
Change in baseline per mmHg	-0.04 [-0.14, 0.06]	0.03 [-0.09, 0.15]	0.374
True RoP at 17 mmHg (dB/year)	-0.62 [-0.82, -0.47]	-0.51 [-0.68, -0.38]	0.254
% Change in RoP per mmHg	0.33 [0.16, 0.51]	0.13 [-0.12, 0.39]	0.191
Learning (dB/year)	0.56 [0.44, 0.69]		< 0.001*
**HRT–RA**			
Baseline at 17 mmHg (dB)	-0.34 [-0.61, -0.08]	-0.85 [-1.13, -0.60]	0.006
Change in baseline per mmHg	-0.05 [-0.11, 0.00]	-0.05 [-0.12, 0.03]	0.831
True RoP at 17 mmHg (dB/year)	-0.05 [-0.08, -0.03]	-0.01 [-0.09, 0.00]	0.433
% Change in RoP per mmHg	0.59 [0.22, 0.97]	0.06 [-3.42, 2.50]	0.487
Learning (dB/year)	-		-
**OCT-pRNFL**			
Baseline at 17 mmHg (dB)	18.71 [18.58, 18.85]	18.67 [18.52, 18.81]	0.655
Change in baseline per mmHg	-0.04 [-0.07, -0.01]	-0.02 [-0.06, 0.02]	0.344
True RoP at 17 mmHg (dB/year)	-0.06 [-0.10, -0.03]	-0.10 [-0.14, -0.06]	0.176
% Change in RoP per mmHg	0.50 [0.13, 0.91]	0.32 [-0.15, 0.76]	0.540
Learning (dB/year)	-		-

Correlation coefficient (True RoP)		
**VF-HRT**	**VF-OCT**	**HRT-OCT**
0.32 [-0.20, 0.78]	0.58 [0.18, 0.99]	0.52 [0.01, 0.98]

Estimate [95%-Credible interval]

Supplementary Table 2. Model estimates for the effect of intraocular pressure on the true rate of progression (RoP) for each arm of the trial. The baseline represents the estimated mean value when time = 0; this was also allowed to vary by average IOP. GAT = Goldman Applanation Tonometry; VF = Visual Field; MD = Mean Deviation; HRT = Heidelberg Retinal Tomograph; RA = Rim Area; OCT = Optical Coherence Tomography; pRNFL = peripapillary Retinal Nerve Fibre Layer; IQR = Interquartile Range. *For difference from 0 dB/year.

Main model estimates with perimetric Mean Linear Sensitivity

GAT	Baseline at 17 mmHg (dB)	Change in baseline (dB) per mmHg	True RoP at 17 mmHg (dB/year)	% Change in RoP per mmHg	Learning (dB/year)
**VF-MLS**	27.69 [27.50, 27.86]	-0.02 [-0.06, 0.02]	-0.28 [-0.38, -0.20]	7.89 [3.91, 11.84]	0.33 [0.23, 0.44]
**HRT-RA**	-0.61 [-0.78, -0.43]	-0.03 [-0.07, 0.02]	-0.05 [-0.07, -0.03]	13.77 [5.72, 22.49]	-
**OCT-pRNFL**	18.65 [18.56, 18.74]	-0.03 [-0.05, -0.01]	-0.08 [-0.11, -0.06]	8.02 [1.79, 14.42]	-

Correlation coefficient (True RoP)		
**VF-HRT**	**VF-OCT**	**HRT-OCT**
0.25 [-0.31, 0.80]	0.38 [-0.24, 0.90]	0.44 [-0.17, 0.96]

Supplementary Table 3. Model estimates for the effect of intraocular pressure on the true rate of progression (RoP), using Mean Linear Sensitivity (MLS, in dB) instead of MD for the VF. The baseline represents the estimated mean value when time = 0; this was also allowed to vary by average IOP. GAT = Goldmann Applanation Tonometry; VF = Visual

Field; MS = Mean Sensitivity (see Methods); HRT = Heidelberg Retinal Tomograph; RA = Rim Area; OCT = Optical Coherence Tomography; pRNFL = peripapillary Retinal Nerve Fibre Layer; IQR = Interquartile Range.

Models for the temporal optic nerve/nasal visual field

With perimetric Mean Sensitivity (mean of dB sensitivity values)

GAT	Baseline at 17 mmHg (dB)	Change in baseline (dB) per mmHg	True RoP at 17 mmHg (dB/year)	% Change in RoP per mmHg	Learning (dB/year)
**VF-MS**	26.43 [26.11, 26.75]	-0.02 [-0.10, 0.05]	-0.63 [-0.76, -0.51]	7.23 [4.13, 10.55]	0.52 [0.41, 0.64]
**HRT-RA**	-5.33 [-5.58, -5.07]	-0.05 [-0.12, 0.01]	-0.12 [-0.16, -0.08]	3.54 [-3.48, 10.89]	-
**OCT-pRNFL**	18.38 [18.27, 18.49]	-0.03 [-0.05, 0.00]	-0.08 [-0.12, -0.05]	10.19 [3.13, 18.07]	-

Correlation coefficient (True RoP)		
**VF-HRT**	**VF-OCT**	**HRT-OCT**
0.40 [0.03, 0.80]	0.69 [0.35, 0.99]	0.44 [-0.19, 0.97]

Supplementary Table 4. Model estimates for the effect of intraocular pressure on the true rate of progression (RoP), using Mean Sensitivity (MS) for the nasal VF and structural measurements from the temporal optic nerve. The baseline represents the estimated mean value when time = 0; this was also allowed to vary by average IOP. GAT = Goldmann Applanation Tonometry; VF = Visual Field; MS = Mean Sensitivity (see Methods); HRT = Heidelberg Retinal Tomograph; RA = Rim Area; OCT = Optical Coherence Tomography; pRNFL = peripapillary Retinal Nerve Fibre Layer; IQR = Interquartile Range.

With perimetric Mean Linear Sensitivity

GAT	Baseline at 17 mmHg (dB)	Change in baseline (dB) per mmHg	True RoP at 17 mmHg (dB/year)	% Change in RoP per mmHg	Learning (dB/year)
**VF-MS**	28.42 [28.24, 28.60]	-0.02 [-0.06, 0.03]	-0.26 [-0.38, -0.17]	8.88 [3.62, 15.37]	0.30 [0.20, 0.40]
**HRT-RA**	-5.33 [-5.60, -5.08]	-0.05 [-0.11, 0.01]	-0.11 [-0.16, -0.08]	3.51 [-4.55, 10.81]	-
**OCT-pRNFL**	18.36 [18.25, 18.47]	-0.03 [-0.05, 0.00]	-0.08 [-0.12, -0.05]	9.96 [1.31, 19.07]	-

Correlation coefficient (True RoP)		
**VF-HRT**	**VF-OCT**	**HRT-OCT**
0.19 [-0.45, 0.77]	0.49 [0.01, 0.94]	0.37 [-0.40, 0.99]

Supplementary Table 5. Model estimates for the effect of intraocular pressure on the true rate of progression (RoP), using Mean Linear Sensitivity (MLS) for the nasal VF and structural measurements from the temporal optic nerve. The baseline represents the estimated mean value when time = 0; this was also allowed to vary by average IOP. GAT = Goldmann Applanation Tonometry; VF = Visual Field; MS = Mean Sensitivity (see Methods); HRT = Heidelberg Retinal Tomograph; RA = Rim Area; OCT = Optical Coherence Tomography; pRNFL = peripapillary Retinal Nerve Fibre Layer; IQR = Interquartile Range.

Models with floor-compensated structural metrics

With perimetric Mean Deviation

GAT	Baseline at 17 mmHg (dB)	Change in baseline (dB) per mmHg	True RoP at 17 mmHg (dB/year)	% Change in RoP per mmHg	Learning (dB/year)
**VF-MD**	-3.86 [-4.14, -3.56]	-0.02 [-0.09, 0.05]	-0.60 [-0.73, -0.48]	7.50 [4.31, 11.05]	0.58 [0.45, 0.70]
**HRT-RA**	-6.32 [-6.66, -5.99]	-0.04 [-0.12, 0.05]	-0.21 [-0.31, -0.14]	12.67 [5.17, 20.73]	-
**OCT-pRNFL**	13.49 [13.24, 13.73]	-0.07 [-0.12, -0.01]	-0.37 [-0.48, -0.28]	8.90 [3.82, 14.48]	-

Correlation coefficient (True RoP)		
**VF-HRT**	**VF-OCT**	**HRT-OCT**
0.56 [0.17, 1.00]	0.75 [0.48, 0.99]	0.69 [0.27, 1.00]

Supplementary Table 6. Model estimates for the effect of intraocular pressure on the true rate of progression (RoP), using MD and floor-compensated structural metrics. The baseline represents the estimated mean value when time = 0; this was also allowed to vary by average IOP. The estimated baselines for the structural metrics differ from the values in the main **Table 3** because of the removal of the structural floor. GAT = Goldmann Applanation Tonometry; VF = Visual Field; MS = Mean Sensitivity (see Methods); HRT = Heidelberg Retinal Tomograph; RA = Rim Area; OCT = Optical Coherence Tomography; pRNFL = peripapillary Retinal Nerve Fibre Layer; IQR = Interquartile Range.

With perimetric Mean Linear Sensitivity

GAT	Baseline at 17 mmHg (dB)	Change in baseline (dB) per mmHg	True RoP at 17 mmHg (dB/year)	% Change in RoP per mmHg	Learning (dB/year)
**VF-MS**	27.69 [27.50, 27.86]	-0.02 [-0.06, 0.03]	-0.27 [-0.39, -0.19]	8.37 [3.22, 14.10]	0.33 [0.23, 0.44]
**HRT-RA**	-6.31 [-6.65, -5.98]	-0.04 [-0.12, 0.04]	-0.21 [-0.31, -0.14]	13.17 [5.44, 21.74]	-
**OCT-pRNFL**	13.44 [13.19, 13.67]	-0.07 [-0.12, -0.01]	-0.36 [-0.47, -0.27]	9.27 [3.40, 15.40]	-

Correlation coefficient (True RoP)		
**VF-HRT**	**VF-OCT**	**HRT-OCT**
0.54 [0.11, 0.97]	0.57 [0.08, 0.98]	0.70 [0.32, 0.99]

Supplementary Table 7. Model estimates for the effect of intraocular pressure on the true rate of progression (RoP), using Mean Linear Sensitivity (MLS) instead of MD for the VF. The baseline represents the estimated mean value when time = 0; this was also allowed to vary by average IOP. The estimated baselines for the structural metrics differ from the values in the main **Table 3** because of the removal of the structural floor. GAT = Goldmann Applanation Tonometry; VF = Visual Field; MS = Mean Sensitivity (see Methods); HRT = Heidelberg Retinal Tomograph; RA = Rim Area; OCT = Optical Coherence Tomography; pRNFL = peripapillary Retinal Nerve Fibre Layer; IQR = Interquartile Range.

With matched structure-function mapping (temporal optic nerve, nasal visual field)

GAT	Baseline at 17 mmHg (dB)	Change in baseline (dB) per mmHg	True RoP at 17 mmHg (dB/year)	% Change in RoP per mmHg	Learning (dB/year)
**VF-MLS**	28.42 [28.23, 28.59]	-0.02 [-0.06, 0.02]	-0.27 [-0.41, -0.17]	8.90 [3.45, 14.77]	0.30 [0.20, 0.41]
**HRT-RA**	-9.82 [-10.24, -9.41]	-0.07 [-0.18, 0.03]	-0.30 [-0.47, -0.19]	5.06 [-5.09, 16.34]	-
**OCT-pRNFL**	13.54 [13.28, 13.83]	-0.05 [-0.11, 0.02]	-0.30 [-0.43, -0.19]	10.25 [2.23, 18.50]	-

Correlation coefficient (True RoP)		
**VF-HRT**	**VF-OCT**	**HRT-OCT**
0.31 [-0.31, 0.94]	0.50 [-0.04, 0.97]	0.53 [-0.21, 0.98]

Supplementary Table 8. Model estimates for the effect of intraocular pressure on the true rate of progression (RoP), using Mean Linear Sensitivity (MLS) for the nasal VF and floor-compensated structural measurements from the temporal optic nerve. The baseline represents the estimated mean value when time = 0; this was also allowed to vary by average IOP. The estimated baselines for the structural metrics differ from the values in the **Supplementary Tables 4 and 5** because of the removal of the structural floor. GAT = Goldmann Applanation Tonometry; VF = Visual Field; MS = Mean Sensitivity (see Methods); HRT = Heidelberg Retinal Tomograph; RA = Rim Area; OCT = Optical Coherence Tomography; pRNFL = peripapillary Retinal Nerve Fibre Layer; IQR = Interquartile Range.
